# Unspecified opioids among opioid overdoses in Oslo, Norway

**DOI:** 10.1186/s13104-022-06022-2

**Published:** 2022-04-09

**Authors:** Zahra Zeineb Iqbal, Thanh Mai Thi Nguyen, Mette Brekke, Odd Martin Vallersnes

**Affiliations:** 1 Faculty of Medicine, University of Oslo, Oslo, Norway; 2 General Practice Research Unit, University of Oslo, Oslo, Norway; 3 Department of General Practice, University of Oslo, Oslo, Norway; 4 Department of Emergency General Practice, Oslo Accident and Emergency Outpatient Clinic, City of Oslo Health Agency, Oslo, Norway

**Keywords:** Opioids, Opiates, Heroin, Buprenorphine, Methadone, Overdose, Poisoning, Naloxone

## Abstract

**Objective:**

Since 2017, an increasing number of opioid overdoses in Oslo, Norway, has been categorized as involving unspecified opioids, as noted in the patient records by the doctor treating the patient. In this study we compare the characteristics of overdoses involving unspecified opioids, long-acting opioids, and heroin. Data on patients presenting with opioid overdose was retrospectively collected from 1 October 2013 to 31 December 2019 at the Oslo Accident and Emergency Outpatient Clinic.

**Results:**

Among 2381 included cases, 459 (19.3%) involved unspecified opioids, 134 (5.6%) long-acting opioids, and 1788 (75.1%) heroin. Overdoses involving unspecified opioids needed longer observation, median 5 h 29 min vs. 4 h 54 min (long-acting opioids) and 4 h 49 min (heroin) (p < 0.001), and had a lower Glasgow coma scale score, median 10 vs. 13 in both the other groups (p < 0.001). Naloxone was given in 23.3% of cases involving unspecified opioids, vs. 12.7% involving long-acting opioids and 30.2% involving heroin (p < 0.001). A larger proportion of patients were transferred to hospital care when unspecified or long-acting opioids were involved compared to heroin, 16.3% and 18.7% respectively vs. 10.1% (p < 0.001). Our results indicate that the category “unspecified opioids” encompasses a substantial proportion of opioids acting longer than heroin.

## Introduction

In Europe there were 1.3 million high-risk opioid users in 2018, and opioids were involved in 82% of drug overdose deaths [1]. The ongoing opioid epidemic in the USA, responsible for about half a million deaths since 1999, has so far come in three waves: first increased prescribing of opioids in the 1990s and overdose deaths from prescription opioids, then an increase in heroin deaths from 2010 following stricter regulation of opioid prescription, finally an increase in overdose deaths related to synthetic opioids from 2013, mainly illegal fentanyl [2, 3].

The main danger of opioid overdose is respiratory depression [4, 5]. Naloxone, an opioid receptor antagonist, is a highly efficient antidote, but has a shorter half-life even than short-acting opioids like heroin [6]. Still, nearly all patients treated with naloxone for a heroin overdose survive without subsequent observation [7, 8]. However, it is not possible to distinguish between different opioids based on the clinical presentation. Accordingly, observation is recommended for two hours after naloxone treatment, to catch any recurring respiratory depression resulting from opioids with longer half-lives [6].

Opioids were involved in 83% of the 275 registered drug overdose deaths in Norway in 2019 [9]. While heroin used to predominate, the last decade has seen an increasing proportion of deaths related to other opioids [9]. In Oslo, the capital city of Norway, most drug overdoses are treated at a primary care emergency outpatient clinic, the Oslo Accident and Emergency Outpatient Clinic (OAEOC). Since 2017, a large proportion of the opioid overdoses treated in this setting have been categorized as involving unspecified opioids, constituting a marked change from previous years [10]. If this category of unspecified opioids to any notable extent were to encompass longer acting opioids than heroin, adhering to the recommended two-hour observation following naloxone administration would be even more important. Hence, in this study we compare the characteristics of overdoses involving unspecified opioids, long-acting opioids, and heroin at the OAEOC.

## Main text

### Methods

Data on patients presenting with opioid overdose at the OAEOC was retrospectively collected from 1 October 2013 to 31 December 2019, using the case definition and data registration tool developed by the European Drug Emergencies Network (Euro-DEN) [11]. We included all cases of overdose related to the recreational use of opioids. Opioid overdoses also involving other drugs or ethanol were excluded.

The diagnosis of drugs taken was based on the clinical assessment noted in the patient records by the doctor treating the patient, in its turn based on the clinical presentation and all available information from the patient and the patient’s companions. The category “unspecified opioid” is used by OAEOC doctors when the patient has an obvious opioid toxidrome (miosis and reduced level of consciousness) and there is no specific information on which opioids have been taken. The OAEOC has limited diagnostic and treatment resources available, and patients needing more intensive treatment are transferred to hospital care [12]. Arterial blood gas and toxicological laboratory analyses are not available. Naloxone is available for intramuscular (main use) and intravenous injection. Naloxone infusion is not given.

Among 5236 opioid overdoses in the inclusion period, 2808 were excluded as other drugs or ethanol also had been taken. The included cases were categorized as involving unspecified opioids (only), long-acting opioids (buprenorphine and/or methadone, including combinations with other opioids except unspecified opioids), and heroin (only). Another 47 overdoses with opioids or combinations of opioids not categorizable into these three groups were excluded.

The three opioid groups were compared on observation time, Glasgow Coma Scale (GCS), vital signs, naloxone administration, and transfer to hospital. Statistical analyses were done using SPSS 27. Continuous variables were compared using Kruskal–Wallis test. Categorical variables were compared using chi-square test, or Fisher’s exact test when > 20% of the cells had an expected count < 5.

### Results

**Fig. 1:**
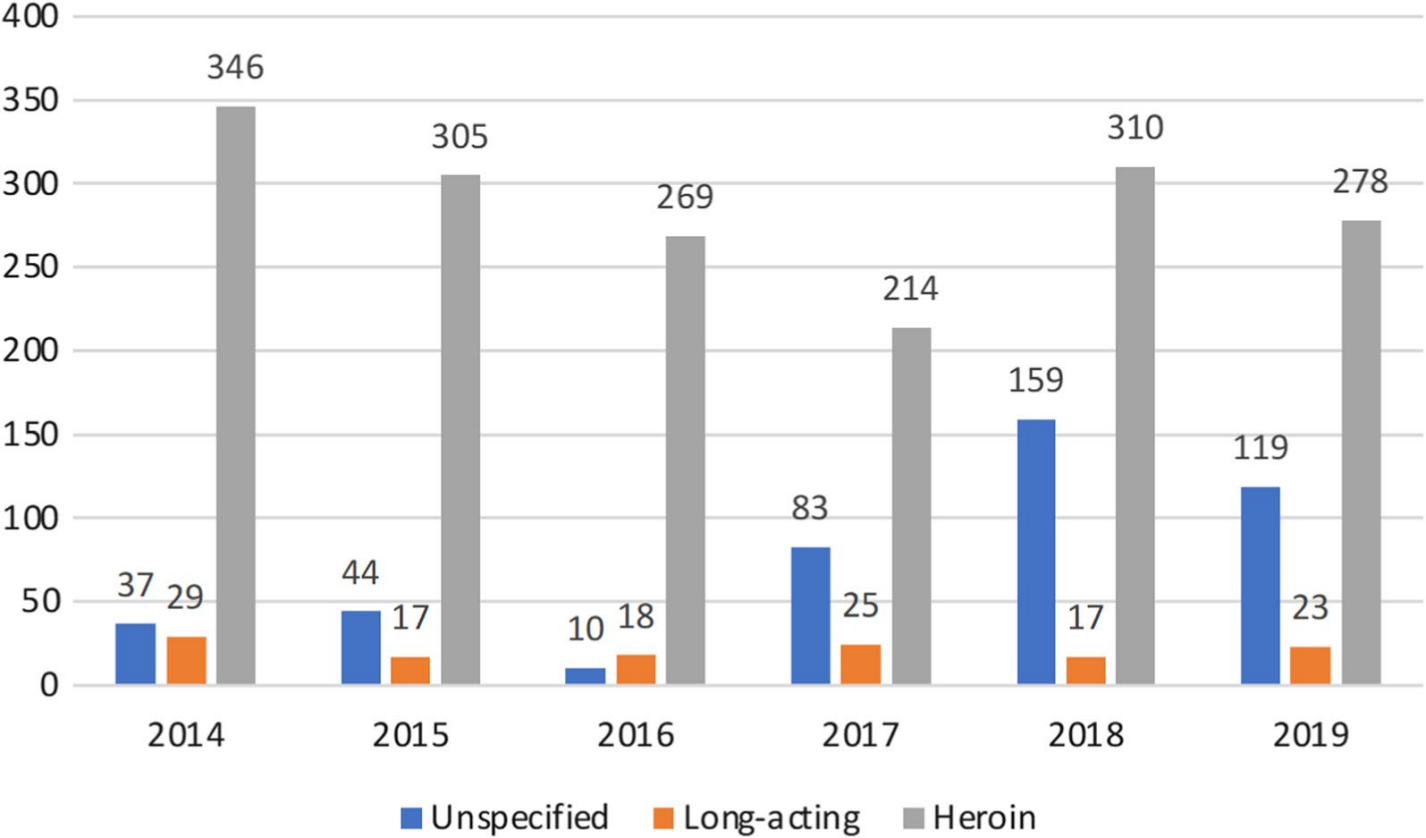
Number of overdoses per year involving unspecified opioids, long-acting opioids, and heroin

In total 2381 opioid overdoses were included. Median age among the patients was 38 years (interquartile range 31–46), 1892 (79.5%) were males. There were 459 (19.3%) cases involving unspecified opioids, 134 (5.6%) involving long-acting opioids, and 1788 (75.1%) involving heroin (Fig. 1).

Cases involving unspecified opioids had longer observation time, median 5 h 29 min vs. 4 h 54 min (long-acting opioids) and 4 h 49 min (heroin) (p < 0.001), and lower GCS than both other groups, median 13 vs. 14 at presentation (p < 0.001) and 10 vs. 13 at the lowest (p < 0.001) (Table 1). In overdoses involving unspecified opioids 23.3% received naloxone compared to 12.7% of cases with long-acting opioids and 30.2% of cases with heroin (p < 0.001). Cases involving unspecified opioids and long-acting opioids were similar to each other but differed from heroin in that a smaller proportion presented with bradypnoea, 17.4% and 19.4% vs. 28.2% respectively (p < 0.001), and a larger proportion was transferred to hospital, 16.3% and 18.7% vs. 10.1% (p < 0.001).

**Table 1:** Characteristics of overdoses involving unspecified opioids, long-acting opioids, and heroin

	Unspecified opioids	Long-acting opioids	Heroin	p-value for comparison across all three groups	p-value for pairwise comparisons		
	n (%)	n (%)	n (%)		Unspecified vs. long-acting	Unspecified vs. heroin	Long-acting vs. heroin
Males	369 (80.4)	99 (73.9)	1424 (79.6)	0.24			
Age *(years)*^*a,b*^	39 (33–46)	44 (36–51)	38 (30–46)	< 0.001	0.001	0.013	< 0.001
Observation time *(h:min)*^*a*^	5:29 (3:49–7:01)	4:54 (2:45–6:48)	4:49 (2:48–6:16)	< 0.001	0.070	< 0.001	0.37
GCS at presentation^a,c^	13 (10–14)	14 (13–15)	14 (13–15)	< 0.001	< 0.001	< 0.001	0.54
Lowest GCS^a,d^	10 (9–13)	13 (11–14)	13 (10–14)	< 0.001	< 0.001	< 0.001	0.46
Bradypnoea *(RR* < *12/min)*	80 (17.4)	26 (19.4)	504 (28.2)	< 0.001	0.69	< 0.001	0.036
Tachycardia *(HR* ≥ *100/min)*	51 (11.1)	14 (10.4)	277 (15.5)	0.024	0.95	0.022	0.15
Bradycardia *(HR* < *50/min)*	9 (2.0)	3 (2.2)	45 (2.5)	0.78			
Hypertension *(BP* ≥ *180 mmHg)*	2 (0.4)	1 (0.7)	10 (0.6)	0.90			
Hypotension *(BP* ≤ *90 mmHg)*	21 (4.6)	5 (3.7)	72 (4.0)	0.85			
Hyperthermia *(temp* ≥ *39.0 °C)*	5 (1.1)	1 (0.7)	16 (0.9)	0.91			
Hypothermia *(temp* < *35.0 °C)*	66 (14.4)	15 (11.2)	149 (8.3)	< 0.001	0.42	< 0.001	0.33
Naloxone treatment	107 (23.3)	17 (12.7)	540 (30.2)	< 0.001	0.011	0.004	< 0.001
Transferred to hospital	75 (16.3)	25 (18.7)	180 (10.1)	< 0.001	0.62	< 0.001	0.003
Total	459 (100)	134 (100)	1788 (100)				

*BP* blood pressure, *GCS* Glasgow coma scale score, *HR* heart rate, *RR* respiratory rate, *temp* temperature

^a^Median (interquartile range)

^b^Missing data for 89 cases (unspecified 49, long-acting 3, heroin 37)

^c^Missing data for 7 cases (unspecified 1, heroin 6)

^d^Missing data for 401 cases (unspecified 26, long-acting 26, heroin 349)

### Discussion

Patients treated for opioid overdose categorized as involving unspecified opioids had a lower level of consciousness and needed longer observation time than other opioid overdose patients, and as many were transferred from outpatient care to hospital admission as when long-acting opioids were involved. This indicates that the category of unspecified opioids among opioid overdoses in Oslo, Norway, encompasses a substantial proportion of opioids acting longer than heroin. The increase in the number of overdoses involving opioids other than heroin is in line with trends seen both elsewhere in Europe and in the USA [1–3] and also in overdose deaths in Norway [9].

The increase in the number of overdoses involving opioids acting longer than heroin also underscores the importance of the recommended two-hour observation following naloxone administration. Patients overdosing on longer acting opioids have a greater risk of recurrent respiratory depression following naloxone treatment and may become in need of repeated naloxone administration. Hence, they should be observed long enough for this potential need to have manifested itself [6]. To avoid acute opioid withdrawal symptoms, which often will antagonize the patient and lead to self-discharge rather than the recommended observation, naloxone should be given intramuscularly (not intravenously) and titrated carefully until the patient breathes adequately [13].

## Limitations

The study was based on clinical diagnosis of toxic agents. Toxicological laboratory analyses could have provided specific information on which opioids constitute the category of unspecified opioids. However, toxicological laboratory analyses are not regularly done at the OAEOC and were not available to us. Though the opioid toxidrome with miosis and reduced level of consciousness is easily recognizable, the lack of laboratory support for the diagnosis of specific opioids is a limitation in our study.

It is possible that some patients were observed longer because they had recurring respiratory depression. However, we did not have information on respiratory function over time. It is highly likely that patients with recurring respiratory depression also had prolonged or recurring reduced level of consciousness, which in itself would lead to longer observation. Hence, we do not think that the lack of this information alters the interpretation of our findings.

Combinations of heroin and undiagnosed benzodiazepines could also be an explanation for the longer observation time seen for unspecified opioids. In a study done at the OAEOC in 2015 with toxicological analysis of saliva samples from patients with recreational drug overdose, benzodiazepines were found in more than half the patients, though not clinically suspected [14]. In the same study, methadone was found in one out of four patients though not clinically suspected [14], confirming that long-acting opioids do occur more frequently than caught by the clinical radar.

## Data Availability

Data are currently not available for sharing. Several manuscripts based on the data set are in preparation. Requests concerning the data may be sent to the corresponding author.

## Abbreviations

BP: Blood pressure
GCS: Glasgow coma scale score
HR: Heart rate
OAEOC: Oslo Accident and Emergency Outpatient Clinic
RR: Respiratory rate; temp: temperature
